# Augmented Decisions: AI-Enhanced Accuracy in Glaucoma Diagnosis and Treatment

**DOI:** 10.3390/jcm14186519

**Published:** 2025-09-16

**Authors:** Marco Zeppieri, Caterina Gagliano, Daniele Tognetto, Mutali Musa, Alessandro Avitabile, Fabiana D’Esposito, Simonetta Gaia Nicolosi, Matteo Capobianco

**Affiliations:** 1 Department of Ophthalmology, University Hospital of Udine, 33100 Udine, Italy; 2 Department of Medicine, Surgery and Health Sciences, University of Trieste, 34127 Trieste, Italy; 3 Department of Medicine and Surgery, University of Enna “Kore”, Piazza dell’Università, 94100 Enna, Italy; 4 Eye Center G.B. Morgagni-DSV, 95125 Catania, Italy; 5 Department of Optometry, University of Benin, Benin City 300238, Nigeria; 6 Faculty of Medicine, University of Catania, 95123 Catania, Italy; 7 Imperial College Ophthalmic Research Group [ICORG] Unit, Imperial College, London NW1 5QH, UK; 8 Eye Clinic, Catania University, Policlinico G. Rodolico, Via Santa Sofia, 95121 Catania, Italy; capobiancoteo@gmail.com

**Keywords:** glaucoma, artificial intelligence, deep learning, augmented intelligence, optical coherence tomography, visual field, teleophthalmology, clinical decision support, explainable AI, federated learning

## Abstract

Glaucoma remains a leading cause of irreversible blindness. We reviewed more than 150 peer-reviewed studies (January 2019–July 2025) that applied artificial or augmented intelligence (AI/AuI) to glaucoma care. Deep learning systems analyzing fundus photographs or OCT volumes routinely achieved area-under-the-curve values around 0.95 and matched—or exceeded—subspecialists in prospective tests. Sequence-aware models detected visual field worsening up to 1.7 years earlier than conventional linear trends, while a baseline multimodal network integrating OCT, visual field, and clinical data predicted the need for incisional surgery with AUROC 0.92. Offline smartphone triage in community clinics reached sensitivities near 94% and specificities between 86% and 94%, illustrating feasibility in low-resource settings. Large language models answered glaucoma case questions with specialist-level accuracy but still require human oversight. Key obstacles include algorithmic bias, workflow integration, and compliance with emerging regulations, such as the EU AI Act and FDA GMLP. With rigorous validation, bias auditing, and transparent change control, AI/AuI can augment—rather than replace—clinician expertise, enabling earlier intervention, tailored therapy, and more equitable access to glaucoma care worldwide.

## 1. Introduction

A recent global meta-analysis estimated that *64.3 million people aged 40–80 years* were living with glaucoma in 2013, a number projected to rise to *≈111.8 million* in 2040, mainly due to population aging and higher urbanization rates [1,2]. Beyond the global estimate of 64.3 million cases in 2013 and the projected rise to 111.8 million by 2040, Tham et al. further showed marked regional disparities: primary open-angle glaucoma is most prevalent in Africa (≈4.2%), while primary angle-closure glaucoma peaks in Asia (≈1.1%). Male sex, African ancestry, and urban residency were significant risk factors, underscoring the uneven distribution of glaucoma burden and the urgent need for region-specific screening and treatment strategies [1]. More recent updates confirm and expand on these projections. In 2020, the Vision Loss Expert Group estimated that glaucoma accounted for 3.61 million blind individuals and 4.14 million with moderate-to-severe visual impairment, representing 8.4% of all global blindness and 1.4% of MSVI, thus consolidating its position as the second leading cause of irreversible blindness worldwide. Notably, while the prevalence of glaucoma-related blindness decreased between 2000 and 2020, the prevalence of moderate visual impairment increased, underscoring the persistent global burden of the disease [3].

Complementing prevalence data, a recent meta-analysis of prospective studies reported a global POAG incidence of 23.46 per 10,000 person-years among adults aged 40–79 years (2022), with an annual cumulative incidence (ACI) of 0.21% for POAG and 0.05% for PACG. Incidence rates were highest in Africa and in low-SDI regions, and the main risk factors included advanced age, male sex, elevated IOP, family history, and myopia [4]. Economic aspects are detailed in Section 4; briefly, while AI-assisted ocular screening can be cost-effective, glaucoma still imposes a major socioeconomic burden and any savings must be weighed against total cost of ownership (infrastructure, cybersecurity, and staff training), particularly in resource-constrained settings [5,6].

Current glaucoma workups combine structural imaging—fundus photographs, red-free RNFL images, optical coherence tomography (OCT), and scanning-laser ophthalmoscopy—with functional testing by standard automated perimetry (SAP) or, more recently, swept-source visual field perimetry and objective pupillography. Yet important limitations remain: (i) inter-observer variability in optic-nerve-head evaluation, (ii) ceiling/floor effects and test–retest noise in SAP, and (iii) the inability of linear trend analyses to capture non-linear or sub-clinical change. These diagnostic gaps have fueled growing interest in data-driven solutions, as summarized by recent reviews on AI in glaucoma care [7,8]. Proof-of-concept studies now show deep learning systems matching specialist performance in detection [9] and outperforming traditional models in progression forecasting [10].

Artificial intelligence (AI)—an umbrella that includes machine, deep, and reinforcement learning methods—has already transformed pattern recognition tasks across ophthalmic imaging [7,11]. Ophthalmology is an ideal test bed because it routinely generates large volumes of high-resolution, standardized images [12]. Since Google’s landmark deep learning system for diabetic retinopathy screening—reported in 2016 by Gulshan et al. and achieving an area under the ROC curve (AUC) of ≈0.99—the glaucoma AI literature has expanded more than ten-fold, as documented by recent systematic reviews [11,13,14]. Dedicated glaucoma reviews have documented an exponential growth in AI-related publications, consolidating taxonomies of deep learning architectures and benchmarking them across public datasets [11]. These surveys emphasize not only the consistent diagnostic accuracy of convolutional neural networks and vision transformers, but also highlight persistent challenges in generalizability, uncertainty estimation, and multimodal integration [11,12]. Early economic modeling—from rural China screening cohorts and from workflow analyses of AI virtual assistants—suggests that smartphone-based triage can be cost-saving, although up-front expenditure for IT infrastructure and training remains substantial in low-resource settings [5,6]. State-of-the-art deep learning architectures, such as convolutional neural networks (CNNs), vision transformers (ViT), and graph neural networks (GNNs), learn hierarchical representations directly from raw pixels or volumetric voxels and consistently outperform feature-engineered machine learning models in both detection and progression tasks [12,13,14,15].

Augmented intelligence (AuI) positions AI as a cognitive aid—not a replacement—for the clinician. In practical terms, AuI dashboards quantify progression risk, highlight image regions that drive the algorithm’s decision, and generate guideline-based management options, while the ophthalmologist retains final authority [16,17,18]. Recent critical appraisals stress that AI deployment in ophthalmology raises six major concerns: bias and clinical safety, cybersecurity, ownership of health data and algorithms, the “black box” problem, medical liability, and the risk of widening inequality in healthcare [19]. Addressing these issues through high-quality local data, explainable models, and equitable access is essential to ensure AI remains a supplement—not a substitute—for physician decision-making. A collaborative “human-in-command” approach is ethically preferable: clinician oversight helps prevent delayed treatment from algorithmic false negatives and service overload from false positives [19]. In the UCSD usability study, the GLANCE CDSS—which shows deep-learning-predicted mean deviation (MD) alongside clinical data—was rated moderately useful (Likert 3.42) and trustworthy (3.27), with a mean system usability scale of 66.1 ± 16.0 (43rd percentile). Clinicians were nevertheless reluctant to space out visual field testing (Likert 2.64), especially in severe cases [16], whereas a recent scoping review found that current saliency map techniques do not yet improve interpretability or clinician trust and should not be used as standalone evidence of model validity [18]. In a large retrospective study of more than 5000 patients, a sequence-aware convolutional LSTM network detected visual field worsening with an AUC of 0.94, substantially outperforming clinician assessment (AUC ≈ 0.63) and remaining robust even when fewer tests were available [20]. More broadly, ophthalmology has been among the earliest adopters of AI owing to its image-rich nature, with major progress reported not only in diabetic retinopathy and AMD but also in glaucoma and cataract [21]. Innovative strategies also integrate human expertise into model training: a U-Net leveraging ophthalmologists’ eye-tracking data while reading glaucoma OCT reports predicted the most-fixated regions with precision ≈ 0.72, highlighting the potential of saliency guided by clinical gaze patterns [22]. Large language models, such as GPT-4, have likewise demonstrated specialist-level performance: in a masked comparison involving 20 knowledge questions and 20 real clinical cases, GPT-4 outperformed glaucoma subspecialists in both accuracy and completeness and matched retina subspecialists for accuracy while exceeding them for completeness, although expert oversight remains essential before clinical use [17].

This narrative review collates peer-reviewed evidence published from January 2019 through July 2025 on artificial and augmented intelligence applications in glaucoma. We structure the synthesis around five themes: (i) diagnostic imaging, (ii) progression prediction and monitoring, (iii) clinical decision support—including large language models, (iv) teleophthalmology, and (v) explainable and federated AI. For each domain, we analyze methodological rigor and DECIDE-AI adherence, translational readiness, regulatory trajectory (EU AI Act and FDA GMLP), and emerging cost-effectiveness data. Nevertheless, early clinical evaluations of AI DSS in glaucoma show suboptimal reporting: a systematic review of 19 studies found low adherence to DECIDE-AI for AI-specific items (~30%) despite good compliance with generic items (~85%), highlighting the need for standardized reporting before wider clinical adoption. We conclude with a roadmap for safe, equitable deployment of AI-enabled glaucoma care [5,13,15,16,17,18,19,20,21,22,23,24]. Due to the significant context-dependency of economic feasibility, we have explicitly compared high-income and low-resource settings, correlating cost-effectiveness with adoption barriers.

## 2. Materials and Methods

This study employed a narrative method to synthesize the most therapeutically pertinent advancements in the utilization of artificial intelligence (AI) and augmented intelligence (AUI) for the diagnosis, risk assessment, and management of glaucoma. To obtain a thorough overview of the subject, we thoroughly searched the biomedical and technical literature in three principal databases: PubMed/MEDLINE (*U.S. National Library of Medicine, Bethesda, MD, USA*), Scopus (*Elsevier, Amsterdam, The Netherlands*), and IEEE Xplore (*Institute of Electrical and Electronics Engineers, New York, NY, USA*). These sources were chosen to encompass both the clinical and computational aspects of AI applications in ophthalmology. The search encompassed papers from 1 January 2019 to 1 July 2025, a timeframe indicative of the proliferation of clinically focused AI research subsequent to significant advancements in deep learning and the enhanced accessibility of ocular imaging datasets. Search methodologies integrated controlled vocabulary with free-text terminology, including “glaucoma,” “artificial intelligence,” “machine learning,” “deep learning,” “augmented intelligence,” “OCT,” “fundus,” “visual field,” “decision support,” and “teleophthalmology.” Boolean operators were employed to enhance sensitivity and specificity. Alongside database searches, we examined the reference lists of recent reviews and meta-analyses to discover other suitable papers that may have been overlooked in our original search. This paper is structured as a narrative review, not a systematic review or meta-analysis, so no methodology has been preregistered, and no quantitative synthesis has been conducted.

Studies were suitable for inclusion if published in peer-reviewed journals, written in English, and concentrated on the application of AI or AUI algorithms—supervised, unsupervised, or self-supervised—pertaining to structural or functional data in glaucoma. Structural data comprised fundus photography and optical coherence tomography (OCT), whereas functional data included perimetry and further evaluations of visual function. We also incorporated studies that reported on the clinical deployment or prospective assessment of such tools, along with those addressing translational issues or real-world integration. Studies detailing algorithm development in isolation, lacking a direct link to glaucoma diagnosis or care, were eliminated unless they exhibited unique methodological breakthroughs with potential therapeutic significance.

The evaluation procedure was performed autonomously by two reviewers possessing competence in ophthalmology and biological artificial intelligence. Initially, titles and abstracts were evaluated for relevance, subsequently followed by a comprehensive evaluation of the entire texts of potentially eligible papers. Disputes were settled by dialogue and agreement. No protocol was preregistered for this review, yet the technique was systematically applied to guarantee transparency and reproducibility. We extracted pertinent information from each selected article, including the algorithm type employed (e.g., convolutional neural networks, ensemble models, and support vector machines), the characteristics of the input data (e.g., fundus imaging, OCT scans, visual fields, or multimodal combinations), the dataset size and provenance (e.g., institutional, public, and multicenter), and essential performance metrics, such as area under the receiver operating characteristic curve (AUROC), accuracy, sensitivity, specificity, and F1 score. Furthermore, we saw if the study incorporated external validation, prospective testing, or exhibited real-world clinical integration. Special emphasis was placed on the interpretability of the algorithms, the inclusion of explainable AI components (e.g., saliency maps and attention mechanisms), and the actionability of the model outputs in a clinical context.

Owing to the significant variability in trial designs, clinical outcomes, and statistical reporting, we refrained from doing a meta-analysis. The gathered data were qualitatively synthesized and categorized according to the principal thematic domains identified in the literature. The topics included image-based diagnostic tools, illness progression prediction, decision support systems incorporating large language models (LLMs), teleophthalmology applications, remote screening, and innovative methods, like explainable and federated AI. This thematic structure enabled us to emphasize overarching difficulties, pinpoint deficiencies in the literature, and investigate intersections between engineering advancements and clinical requirements.

We recognize many limitations intrinsic to our methodology. The review was limited to English-language publications, potentially introducing a language bias and omitting pertinent findings published in other languages. We excluded gray literature, including preprints, conference papers, and unpublished datasets, which may have constrained the incorporation of the latest or preliminary advancements in the field. Due to the narrative format of this review and the lack of a preregistered process, there exists a potential for subjective bias in the selection and interpretation of the literature. We alleviated this risk by implementing a transparent and reproducible search strategy, performing independent screening and data extraction, and concentrating on studies with explicit methodological reporting and clinical significance.

This review offers a comprehensive and organized examination of the utilization of AI and AUI technologies in glaucoma management, highlighting existing capabilities and the translational challenges that must be overcome for these tools to be broadly and safely adopted in clinical settings. Eligible studies were peer-reviewed, English-language articles evaluating AI or augmented intelligence applications in glaucoma—diagnosis, progression monitoring, treatment decision support, or teleophthalmology. We excluded preprints, conference abstracts, gray literature, and studies limited to algorithm development without direct clinical relevance. The databases searched, key terms, and full eligibility criteria are summarized in Table 1.

Beyond eligibility criteria, we qualitatively appraised each study across eight domains:

1. **Validation** (external, multicenter, and prospective).
2. **Calibration and clinical utility** (calibration curves and decision curve analysis).
3. **Reporting** (adherence to DECIDE-AI and TRIPOD-AI, when applicable).
4. **Bias management** (case selection, disease spectrum, imbalances by ethnicity/age/myopia, data leakage, and overfitting).
5. **Robustness** (subgroup and sensitivity analyses).
6. **Transferability** (clinical domain, device/vendor, and image quality).
7. **Safety** (uncertainty estimates and clinician hand-off).
8. **Evidence of impact** (clinical or process endpoints).

Given the heterogeneity of the evidence base, we synthesized judgments narratively, classifying overall risk of bias as high, moderate, or low.

## 3. Results

To improve transparency and comparability, when possible, each study summary includes (i) sample size and origin, (ii) validation level (internal, external, or prospective), (iii) spectrum bias risk and case enrichment, (iv) calibration or decision curve reporting, and (v) compliance with AI-specific reporting standards, when relevant (DECIDE-AI/TRIPOD-AI). Consequently, performance indicators have been reported in conjunction with methodological constraints rather than independently (refer to Table 2). Subsequently, we have summarized key performance results and evaluated methodological rigor and generalizability, correlating the discussion with Table 2, Table 3 and Table 4, which delineate advantages and limitations.

### 3.1. Diagnostic Imaging

Fundus photo AI. Liu et al. developed a ResNet-34 system trained on 241,032 color photographs from the Chinese Glaucoma Study Alliance. On the Handan population set the network achieved AUC 0.964, sensitivity 91%, and specificity 92.6% [13]. In subsequent work targeting low-bandwidth contexts, Hemelings et al. showed that a ResNet-50-based CNN achieves AUC 0.94 on disk-centered images and maintains clinically useful performance (AUC ≈ 0.88) even after complete papilla masking, demonstrating that the network exploits peripapillary cues beyond the ONH [29]. Pascal et al. then introduced a multi-task U-Net (17M parameters, ≈3.5× smaller than single-task models) that simultaneously segments the disc, cup, and fovea and detects glaucoma, achieving AUC ≈ 0.97 on the REFUGE dataset [30]. A recent review by Girard and Schmetterer reports that early vision transformer backbones are emerging as a promising and parameter-efficient alternative for the same task [12].

OCT-based AI. Medeiros et al. pioneered a machine-to-machine CNN that regresses OCT-derived average RNFL thickness directly from fundus photographs (ResNet-34), achieving r ≈ 0.83 and MAE ≈ 7 µm—the first clear proof of cross-modal learning [26]. Since then, three complementary OCT strategies have matured, and three main OCT-based AI strategies have emerged. The first, RNFLT2Vec, involves pretraining an encoder on more than 42,000 RNFL thickness maps. This approach reached an AUC of around 0.90 for distinguishing glaucoma from controls while reducing manual annotation needs by over 50%, thanks to automated artifact correction [37].

The second approach relies on fully supervised 3D U-Net models, which, in the studies summarized by Girard and Schmetterer, achieved Dice scores between 0.91 and 0.94 for RNFL and GCC segmentation, with classification AUCs up to 0.96 [12].

Finally, vision transformer-based fusion models that combine volumetric OCT data with parameters such as ILM curvature and lamina cribrosa depth have reported AUCs of 0.94 for early glaucoma detection, with the greatest improvements observed in highly myopic optic discs [14].

Multimodal pipelines are now emerging: a late-fusion graph neural network that ingests SAP probability plots, RNFL thickness maps, and optic disc photographs outperformed a single-modal CNN (AUC 0.97 vs. 0.92) while cutting false positives by ≈23% in the 2000-eye test set reported by Martucci et al. [14]. Key strengths include large datasets with multiple external validations and consistently high performance (AUC ~0.90–0.97) on both fundus and OCT images. Several studies explicitly compared AI systems with clinical benchmarks, adding relevance to their findings. Recurrent limitations include spectrum bias from enriched case selection, declining performance on multi-ethnic or low-quality images, calibration rarely reported, metrics calculated at the image rather than patient level, and limited analyses in challenging subgroups (high myopia, small discs, and NTG). Major gaps remain, such as prospective multicenter, multi-vendor trials, benchmarking against clinical outcomes and operational costs, and protocols addressing domain shifts from new scanners or workflows.

In fundus photography and OCT pipelines, diagnostic AUCs have aligned within the 0.90–0.97 range, demonstrating consistent improvements through multimodal fusion and 3D or transformer architectures. The most consistent failure modes have included spectrum bias from enriched case series, attenuation in multi-ethnic or low-quality photos, and vendor-related domain shifts. Reported calibration has frequently been inadequate, indicating the necessity for recalibration before deployment. Collectively, these patterns suggest that image-only classifiers have advanced for triage and referral, although patient-level calibration and vendor transferability continue to pose obstacles. 3 summarizes the primary advantages and limitations of representative imaging models, facilitating direct comparison across datasets and validation tiers.

External validation was present in 5 out of 14 studies, with sample sizes ranging from 3000 to 80,000 eyes and prevalence rates between 12% and 42%. The risk of spectrum bias was moderate due to enriched case-control datasets. Class imbalance was primarily addressed through augmentation and oversampling techniques. The handling of missing data was not reported. Calibration was documented in three studies, accompanied by calibration plots; otherwise, it was not reported. Clinical utility, assessed via decision curve analysis, was mentioned in only one study. Leakage controls were inconsistent, with some employing subject-wise splits while others utilized image-level splits. Adherence to the TRIPOD-AI reporting standard was not reported in most studies but was explicitly stated in two studies.

### 3.2. Progression Prediction and Monitoring

Longitudinal modeling. Sabharwal et al. trained a convolutional LSTM on sequences of SAP deviation maps: in an external test set, the model achieved an AUC of 0.94, outperforming a mixed-effects linear model (AUC 0.82; *p* < 0.001). Even when the six most recent VFs were withheld, the model still detected worsening with an AUC 0.78 [20]. On a separate longitudinal dataset, Yousefi et al. presented a visual field progression index obtained using machine learning techniques that integrates spatial information from the entire grid; in retrospective analyses, the index signaled progression earlier than traditional global, regional, and point-wise approaches, highlighting the potential for reducing detection times [31].

Extending the concept to structural endpoints, Wang et al. trained a multimodal model based on vision transformer (ViT) combining, from a single baseline visit, OCT RNFL thickness maps (12 × 12), total visual field deviations 24-2 realigned in the same grid, and clinical demographic variables. The algorithm predicted the need for incisional surgery within 3 months with AUROC 0.92 (95% CI 0.88–0.96), remaining ≥0.80 for horizons up to 3 years [25]. Finally, Ha et al. extracted, through a convolutional auto-encoder, features from a pair of baseline images (optic disc photograph + red-free RNFL photograph) and combined them with 15 clinical demographic variables in an XGBoost model: the algorithm predicted the conversion of normotensive suspects to normotensive glaucoma with AUC 0.99 and estimated the time-to-conversion with MSE 2.24 years, providing individual risk curves useful for shared counseling [38].

Progression detection in longitudinal data. Mandal et al. trained a weakly supervised CNN-LSTM (Noise-PU) model on five peripapillary OCT sequences: at a specificity set at 95%, the algorithm identified structural progression in 49.8% of the series versus 28.4% obtained by OLS linear regression on RNFL profiles, with a relative increase in sensitivity of ≈75% thanks to the correction for age-related thinning [39]. Building on self-supervision, Shi et al. pretrained a vision transformer encoder (RNFLT2Vec) on over 42,000 unlabeled RNFL thickness maps. With fine-tuning on less than 5% of labeled data, the model achieved an AUC of approximately 0.90 in glaucoma/control discrimination on an external test set, while reducing annotation effort by about 50% thanks to automatic artifact correction [37].

Continuing on the OCT analysis line, Mariottoni et al. trained a CNN that, starting from two RNFL profiles (baseline + follow-up, 768 points each) and the time elapsed, estimates the probability of progression: the model reached AUC 0.94, with sensitivity 87% and specificity 86%; with the same specificity (92%), the sensitivity increased from 46% (linear global trend) to 76%, surpassing both the global and sectoral analyses [40]. Extending sequence modeling to color fundus photographs, Lee et al. applied the machine-to-machine (M2M) model—trained to estimate RNFL thickness from fundus photographs—on 1072 suspect eyes followed for 5.9 ± 3.8 years: in a joint longitudinal survival model, every 10 µm of predicted RNFL thickness decrease at baseline increased the risk of glaucoma conversion by 56% (HR 1.56, 95% CI 1.33–1.82), while every 1 µm/year of accelerated thinning nearly doubled the risk (HR 1.99, 95% CI 1.36–2.93). The model explained approximately 51% of the variance in time-to-event, outperforming traditional clinical models [32].

Home rebound tonometry systems, such as iCare HOME, have been proposed as a continuous source of pressure profiles in the cloud. Early studies are evaluating their concordance with Goldmann tonometry and their usefulness as input for future AI-based predictive models [21]. Longitudinal and multimodal models show promise in anticipating progression while maintaining sensitivity with fewer tests, and some studies provide interpretability through methods such as SHAP. However, most evidence comes from retrospective, single-center studies, with heterogeneous definitions of progression, little attention to lead time or net benefit, and a non-negligible risk of temporal data leakage. Key gaps include pragmatic RCTs on follow-up schedules, evaluations with decision curve analysis and therapy-modifying impact, and dedicated analyses in complex phenotypes (PXF, PACG, and NTG).

Sequence-aware models have consistently surpassed static or linear trend methodologies and have maintained valuable sensitivity even with a reduced number of tests. Discrepancies among studies have primarily arisen from inconsistent progression labels, varying censoring practices, and insufficient focus on decision curve or net-benefit assessments. Significant improvements have been noted when structural and functional data are integrated at baseline; nevertheless, the definitions of ‘rapid progression’ and the timelines for risk prediction have differed. These factors have emphasized the necessity for standardized endpoints and forward-looking evaluations that connect model outputs to treatment-altering decisions. Table 2 presents a comparative analysis of longitudinal and multimodal methodologies, detailing their validation status and associated risks (temporal leakage, heterogeneous labels).

External validation is absent in the majority of studies, present in two out of nine. Sample sizes range from 500 to 5000 patients, with progression prevalence between 18% and 30%. The risk of spectrum bias is moderate due to clinic-based samples and high baseline risk, while class imbalance is generally unaddressed, with only two studies applying weights. Most studies utilized complete-case analysis for missing data, with imputation not reported. Calibration was documented with expected/observed ratios in one study, while decision curve analysis was not reported in any study. Leakage controls were inadequate in several instances, with likely temporal leakage, while three studies employed appropriate longitudinal splits. Adherence to the TRIPOD-AI reporting standard was not reported across all studies.

### 3.3. Clinical Decision Support and LLMs

In detail, ten clinicians assessed six cases (eleven eyes) with GLANCE. Perceived trust and usefulness of the predicted MD were 3.27 and 3.42/5, willingness to reduce visual field frequency was 2.64/5, and the overall SUS score was 66.1 ± 16.0 [16]. Large language models show complementary promise: in a masked, single-center study, Huang et al. found that GPT-4 achieved higher mean rank scores for accuracy and completeness than glaucoma subspecialists (*p* < 0.001) and equaled retina subspecialists for accuracy while surpassing them for completeness (*p* = 0.005) [17]. Delsoz et al. tested ChatGPT-3.5 on 11 published clinical reports of primary and secondary glaucoma: the model provided the correct provisional diagnosis in 8 out of 11 cases (≈73%), a performance comparable to 2 of the 3 senior residents (73% and 73%) and superior to one (55%). ChatGPT (GPT-4, OpenAI March, 2023) also produced a wider range of differential diagnoses, while remaining consistent with the clinical picture [33]. In a prospective study of 26 complex glaucoma cases, Zhang et al. compared GPT-4.o with 3 ophthalmologists: on the 10/10 accuracy scale, GPT-4o scored 5.5 points versus 6.8–8.0 for clinicians (*p* < 0.001), while for the accuracy of differential diagnosis it equaled the 2 most experienced specialists (~7.6/10) and, above all, provided the most complete list (4.1/6 vs. 2.8–3.8). The authors conclude that GPT-4o cannot replace human expertise for primary diagnosis but can serve as a useful support in complex cases [34]. Table 1 summarizes key performance metrics and contextual features of representative AI models in glaucoma diagnosis, progression monitoring, and clinical decision support published between 2019 and 2025. Clinical decision support tools are perceived as useful, particularly in complex cases, and large language models (LLMs) offer broad coverage of medical knowledge. Yet, limitations are notable: small sample sizes, usability-focused rather than outcome-driven studies, risks of hallucination, lack of calibration and uncertainty quantification, and absence of formalized human-in-the-loop pathways. Future work should prioritize trials on process outcomes (e.g., referral times and inappropriate referrals), patient-level clinical outcomes, medico-legal implications, and integration with EHR systems.

**Figure 1:**
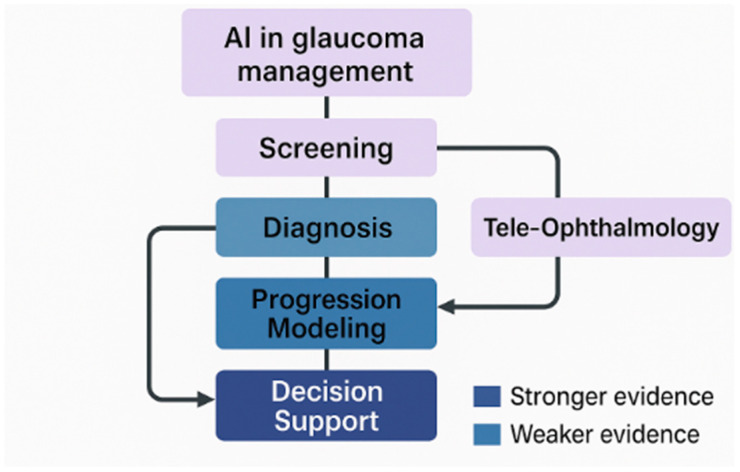
Conceptual evidence map of AI applications in glaucoma care.

Decision support dashboards and LLM-based tools have demonstrated efficacy in intricate cases and knowledge tasks; however, limited sample sizes, insufficient calibration or uncertainty quantification, and the potential for hallucination have restricted generalizability. Consistent patterns have encompassed clinicians’ perceived usefulness and performance, nearing subspecialist precision on organized problems; nevertheless, reports have infrequently addressed outcome-level influence, medico-legal governance, or human-in-the-loop routes. The identified shortcomings have highlighted the necessity for prospective studies that evaluate processes and patient outcomes, as well as standardize escalation and hand-off protocols. The ‘Limitations’ column in Table 2 has highlighted issues related to calibration, uncertainty reporting, and governance deficiencies for decision-support tools and LLMs. While Figure 1 summarizes the evidence landscape across screening, diagnosis, progression modeling, decision support, and tele-ophthalmology.

External validation is lacking (pilot or proof-of-concept only). Sample sizes range from n = 50 to 500 case vignettes or EHR records, with prevalence not reported. There is a high risk of spectrum bias (selected challenging cases), class-imbalance management is not reported, missing data are not reported, calibration is not reported, clinical utility is not reported (no outcome-level evaluation), and leakage controls are not applicable (static test sets only). Adherence to reporting standards STARD-AI/DECIDE-AI is not reported.

### 3.4. Teleophthalmology and Remote Monitoring

In a prospective trial on 243 patients (111 with glaucoma), Rao et al. validated an offline “on-device” algorithm integrated into a fundus-on-phone. Compared to the complete specialist evaluation, it achieved a sensitivity of 93.7% and a specificity of 85.6% (true-negative rate in non-glaucoma patients 94.7%). On the identical series of images, the AI detected 94% of glaucoma, while three blinded specialists detected only 60% [27], and the Medios Glaucoma AI tool achieved 91.4% sensitivity and 94.1% specificity in a real-world, specialist-confirmed clinical cohort of 213 patients [28]. For at home follow-up, Dolar-Szczasny et al. reported that home OCT devices demonstrated high diagnostic accuracy, with sensitivity up to 98.5% and specificity up to 98.8% in some AI-integrated pilot programs, and strong correlation (r = 0.90–0.998) with in-office OCT for central subfield thickness [35]. Herbert et al. demonstrated that a multimodal deep learning model combining early VF, OCT, and clinical data predicted rapid glaucoma worsening with high accuracy (AUROC up to 0.87), improving performance compared to logistic regression models [36]. Current evidence shows strong offline and on-device performance, with feasibility demonstrated in resource-limited settings, and home OCT has shown high concordance with clinic-based imaging. Nonetheless, important limitations persist. Studies are geographically concentrated (mainly India and selected centers), performance is highly dependent on image quality, most studies are pilot projects with short follow-up, and adherence data for home use are limited. Research priorities include multicenter community-based trials, cost-effectiveness analyses beyond Asia, and structured referral pathways addressing service burden.

Offline smartphone classifiers exhibit elevated sensitivity and specificity for referable glaucoma in clinics catering to low-resource communities, whereas home OCT initiatives demonstrate robust technical concordance with clinic-based imaging. Discrepancies have emerged due to spatial concentration of evidence, brief follow-up periods, and dependence on image quality. The real-world impact has consequently depended on organized referral pipelines, retesting procedures, and adherence assistance, which have not been consistently documented (Table 3 and Table 4).

External validation was present in three out of seven studies (multi-site community deployment). Sample sizes ranged from 1000 to 25,000 screened individuals, with glaucoma prevalence between 3% and 8%. The risk of spectrum bias was low to moderate (population-based), and class imbalance was addressed through oversampling or weighting in two studies, while the others employed no such methods. Missing data were variably excluded, with imputation not reported. Calibration was documented in two studies with the calibration slope, decision curve analysis was absent, and leakage controls were adequate through subject-wise partitioning. Adherence to the reporting standard STARD-AI was stated in one study, with no reports otherwise.

### 3.5. Explainable and Federated AI

Explainable AI (XAI) techniques are designed to make an algorithm’s reasoning transparent and clinically interpretable [19,41]. In glaucoma image classifiers, pixel-based saliency methods—Grad-CAM, integrated gradients, and SmoothGrad—are now routinely appended to highlight optic disc or RNFL regions that drive the output [18,22]. A 2024 systematic review by Wong et al. analyzed 24 ophthalmic studies that used saliency maps and concluded that there is little empirical evidence that such overlays improve diagnostic accuracy, speed, or user confidence. They emphasized technical limitations (low resolution, scale sensitivity, and spurious correlations) and called for standardized evaluation frameworks before saliency maps are adopted clinically [18]. Wong et al. warn that, in the absence of standardized assessments, saliency maps can mislead the user and should, therefore, be used with caution [18]. Complementing these approaches, Tian et al. trained a U-Net on the eye fixations of glaucoma experts, successfully predicting with high accuracy [22].

Sriwatana et al. show that, after artefact correction, the DINO-ViT model achieves an MAE of 3.46 dB (0.15 dB less than the same model trained on uncorrected maps) and outperforms all CNNs, including ResNet-34, and that Grad-CAM/attention maps focus on the cup, neuro-retinal margin, and wedge-shaped RNFL defects, consistent with the predicted scotomas [41]. Such built-in saliency may help manufacturers comply with the EU MDR/EU AI Act requirement that AI outputs be traceable to a “human understandable rationale” [24].

In a federated study involving seven centers in Hong Kong, Singapore, and the United States, Ran et al. trained three 3D CNN networks on OCT images without exchanging raw data: accuracy across sites ranged from 78% to 98%, significantly reducing the inter-center gap [15]. Koornwinder et al. demonstrate that a TabNet model that combines EHR data and RNFL OCT parameters predicts surgery within 12 months with an AUROC of 0.832, outperforming XGBoost [42].

Although no clinical implementations have been reported yet, schemes combining federated learning and explainable outputs have been proposed for resource-constrained contexts. Yuksel Elgin et al. propose a conceptual framework for equitable glaucoma progression prediction, adaptable to a hub-and-spoke model spanning rural, community, and tertiary sites. While still theoretical, the framework envisions lightweight deployment, privacy-preserving training methods, such as federated learning, and interpretable outputs to support clinician trust [43].

Remaining gaps: (i) Evidence on clinician acceptance remains limited, particularly in terms of long-term adoption and deployment across diverse healthcare settings. Yuksel Elgin et al. identify this as a key implementation challenge [18,43]. (ii) No consensus quantitative metric yet exists for saliency map quality, making cross-study comparison difficult [19,22]. (iii) The regulatory framework on how to validate and reauthorize federated models is still evolving. In the United States, the debate concerns precisely how to manage continuous updates without repeating the entire authorization process [24]. Addressing these gaps will require larger human factor trials, standardized XAI benchmarks, and routine bias audits so that transparency genuinely translates into safer glaucoma care. Early evidence suggests model attention often overlaps with clinically meaningful regions, and federated learning reduces inter-center variability without direct data sharing. However, the evidence base remains thin: saliency maps have not been shown to improve accuracy or trust, XAI metrics are not standardized, FL infrastructure is challenging to implement, and regulatory pathways for continuously updated models remain undefined. Future priorities include shared XAI benchmarks, routine bias audits, and maintainability and change control studies aligned with EU AI Act and FDA GMLP frameworks.

Saliency and attention methodologies frequently coincide with clinically significant areas; however, empirical evidence demonstrating their enhancement of diagnostic accuracy or trust remains scarce. Federated learning has maintained privacy while nearing the accuracy of centralized training, yet infrastructure requirements and ambiguous reauthorization processes for continuously updated models remain challenges. These discoveries underscore the necessity for collaborative XAI benchmarks, regular bias assessments, and studies on maintainability and change control in accordance with the EU AI Act and FDA GMLP standards (Table 4).

External validation was present in two out of six federated learning studies, but absent in studies focused solely on explainable artificial intelligence (XAI). Sample sizes range from 2000 to 12,000 eyes, with prevalence not reported. The risk of spectrum bias is moderate due to the prevalence of single-vendor datasets, class imbalance is addressed through augmentation in most cases, missing data are not reported, calibration is documented with plots in one study, clinical utility remains unreported, and leakage controls are generally adequate, employing subject-wise partitioning. Adherence to the TRIPOD-AI reporting standard is not specified.

### 3.6. Emerging Insights

A quick PubMed, Scopus, Web of Science, and engineering-focused indexes (IEEE Xplore) search (Title/Abstract + MeSH) restricted to the past five years—31 July 2020 through 31 July 2025—using the string *glaucoma* AND (*artificial intelligence* OR *machine learning* OR *deep learning*) retrieved >350 abstracts. Among the most clinically relevant developments, two prospective studies from tertiary centers in India showed that **smartphone-based AI screening** can reach overall accuracies between 89% and 92%, with very high sensitivity—about 94% overall and up to 96% for advanced disease. In the study by Rao et al., sensitivity was 93.7% with specificity of 85.6%, while Senthil et al. reported a sensitivity of 91.4% and specificity of 94.1% [27,28].

In the context of **progression risk modeling for ocular hypertension**, neural networks trained on nine clinical parameters achieved validation accuracies up to 75% for predicting PSD, risk score, and cup-to-disc ratio [44]. Meanwhile, a lightweight CNN trained on 15,000 Humphrey visual field PDF reports reached 100% accuracy for numerical data extraction and ≥98.6% for metadata recognition, effectively **automating visual field digitization and workflow integration** [45].

An **equity-focused framework** called AI GLOBE was also introduced to bring specialist-level progression prediction to rural and urban clinics alike, using federated learning and interpretable outputs [43].

On the topic of **disease staging**, analysis of pattern deviation plots achieved an overall F1 score of 96.8% across normal, early, moderate, and advanced glaucoma, indicating that AI can support not only detection but also standardized severity grading [46].

Finally, a **ChatGPT-4** vision **pilot study** analyzing 300 fundus images reported a peak accuracy of 81% on RIM-ONE, with lower performance on ACRIMA (68%) and ORIGA (70%). These results highlight the variability across datasets and underscore the ongoing need for specialist oversight before routine clinical deployment [47].

Taken together, these findings reflect a transition from proof-of-concept studies to clinically actionable tools, while emphasizing the need for rigorous multicenter validation, systematic bias mitigation, and clear regulatory guidance [11,19].

Overall, the evidence across domains points in the same direction. In imaging, both fundus- and OCT-based systems now deliver AUCs stably in the 0.90–0.97 range, confirming that automated image analysis has reached a level of reliability that matches clinical needs. When disease progression is modeled longitudinally rather than with static or linear methods, detection becomes earlier and more sensitive, and multimodal pipelines—bringing together visual fields, OCT, and clinical data—provide another clear performance gain. Decision support tools and large language models are not a substitute for expertise but already show they can complement the clinician, especially in complex or time-pressured contexts. Teleophthalmology studies demonstrate that high-performing algorithms can be deployed offline or on smartphones, extending access to settings where subspecialty care is scarce. Finally, the growing use of explainable and federated approaches shows that the field is actively moving toward transparency, equity, and safe scalability. Taken together, these results indicate that AI in glaucoma is no longer confined to proofs of concept but is maturing into tools with tangible clinical utility—while still facing shared challenges of generalizability, interpretability, and integration into everyday practice. Table 3 summarizes pertinent studies on AI. Table 4 lists the cross-domain synthesis of strengths, weaknesses, risks, maturity of evidence, and future priorities in AI for glaucoma.

Studies reported in this paper have several limitations. We have emphasized methodological constraints, such as small or single-center cohorts, lack of external or prospective validation, risks of spectrum bias, image-level rather than patient-level metrics, and inadequate reporting of calibration and net benefit. The aforementioned issues have impacted the dependability and transportability of the presented measures, as indicated beside each result summary. These emerging trends have both reinforced and contextualized the domain-specific patterns outlined in Section 3.1, Section 3.2, Section 3.3, Section 3.4, Section 3.5 and have prompted the cross-cutting considerations elaborated in Section 4.

## 4. Regulatory, Ethical, and Economic Considerations

The FDA’s 2018 authorization of the autonomous IDx DR system for diabetic retinopathy screening is often cited as a precedent, yet no glaucoma-specific autonomous SaMD has so far obtained clearance. In Europe, glaucoma decision support tools may obtain a CE mark as class IIa software, but the EU Medical Device Regulation requires both prospective clinical evidence and ongoing post-market surveillance. Consolidated guidance is still emerging: the forthcoming EU AI Act likewise requires providers of high-risk medical AI systems to embed rigorous version tracking and change control procedures within their AI quality management system, ensuring full traceability across successive algorithm updates [24].

Economic indicators have varied significantly among health systems. In low-resource settings, offline, smartphone-based triage and teleophthalmology have proven to be cost-effective compared to the absence of screening and have provided practical accessibility; however, the overall expenses for population-level imaging, confirmatory testing, and ongoing follow-up have remained significant. In high-income systems, incremental cost utility has relied on local willingness-to-pay thresholds, the availability of reimbursement codes, and the extent to which AI-assisted paths have diminished needless visits or expedited appropriate referrals. The total cost of ownership—comprising IT infrastructure, data security, model maintenance, and staff training—has tempered overall savings and influenced the transition of pilot success into sustained adoption across various settings. These context-specific limitations elucidate why favorable cost-effectiveness ratios have not led to consistent implementation, highlighting the necessity for implementation trials that document both clinical outcomes and subsequent service metrics, including referral volume, retest rates, and waiting time distributions.

Ethical discussion centers on liability, algorithmic fairness (e.g., potential bias against small disc hyperopes, high myopes, or normal tension glaucoma), and data privacy. Recent position papers call for mandatory bias audits and model transparency standards before large-scale deployment [19,24]. From an economic perspective, a cost utility study in rural China estimated that AI-assisted fundus screening costs ≈ USD 6100 per QALY, well below the willingness-to-pay thresholds in many low- and middle-income settings [5]. Implementation analyses also note that infrastructure, cybersecurity, and staff training costs can offset part of these savings, particularly when virtual assistant platforms are introduced into primary eye care workflows [6]. Overall, robust clinical evidence, bias mitigation, and harmonized regulatory pathways remain prerequisites for safe and economically viable adoption of glaucoma AI.

Recent economic modeling from a rural China program shows that, although AI-assisted ocular screening can be cost-effective, the absolute outlays for population-wide screening and follow-up care remain substantial for low-resource health systems [5]. These findings are consistent with broader assessments highlighting glaucoma as not only a leading cause of irreversible blindness but also a major socioeconomic burden, where treatment to lower intraocular pressure often requires life-long polypharmacy or surgery without guaranteeing disease arrest [2]. In this context, AI-assisted strategies may represent one of the few cost-effective options for scaling up screening in resource-constrained environments [5,6]. In parallel, a narrative review on AI virtual assistants in primary eye care highlights that any savings gained through automation must be balanced against the upfront expenses for IT infrastructure, cybersecurity, and staff training, which can further strain resource-constrained clinics [6].

Supporting evidence from high-income settings confirms this trend: a Dutch Markov model of repeated AI-based fundus photo screening (every 5 years in individuals aged 50–75) estimated an ICER of ≈EUR 19,311 per QALY gained, with a 51.2% probability of being below the EUR 20,000 willingness-to-pay threshold. This strategy detected 1.6× more cases and reduced visual impairment by 0.8 months per screened individual [48].

Similarly, a modeling study in China found that AI-enabled telemedicine screening dominated “no screening” in rural areas and achieved an ICER of only USD 2567 in urban settings, outperforming both conventional and non-AI telemedicine strategies [49].

More broadly, a 2025 systematic review across multiple clinical domains (including ophthalmology) reported that AI interventions generally improve QALYs and reduce costs but warned that many economic evaluations underreport infrastructure and indirect costs, leading to possible overestimation of benefits. The authors call for more rigorous, context-specific economic studies to validate these promising trends [50].

## 5. Discussion

### 5.1. Overall Synthesis of Findings

In this review, we have aggregated convergent patterns, highlighted discrepancies, and outlined shared constraints based on the integrative remark at the conclusion of each results domain, leading to concrete implementation steps. AI systems for glaucoma diagnosis already show high performance on very large, multi-ethnic datasets: on over 240,000 fundus photographs and OCT scans, several models have reported AUROCs between 0.94 and 0.99, with sensitivities up to 96% [9,13,26]. In a retrospective head-to-head study, these algorithms even matched or surpassed 13 clinicians, including glaucoma specialists [9]. Models incorporating the temporal dimension are also able to intercept functional deterioration earlier. Sabharwal et al. demonstrated that their spatiotemporal network maintained an AUROC of 0.78 even when removing the six most recent perimetries, a sign of the ability to detect progression with less data (even without quantifying lead time) [20]. Similarly, Yousefi et al.’s progression index identified 25% of eyes deteriorating approximately 1.7 years earlier than the traditional mean deviation slope [31].

A dashboard that combines structured EHR data with deep learning predictions of visual field progression was judged usable by clinicians, who noted that the AI output could guide—but not replace—their management choices [16]. Large language model chatbots (GPT-4/ChatGPT) (version GPT-4 OpenAI, San Francisco, CA, USA; released on March, 2023) matched or even surpassed glaucoma subspecialists in answering clinical questions and managing real cases [17,33]. Offline AI embedded in smartphone-based fundus cameras achieved 93.7% sensitivity and 85.6% specificity for referable glaucoma in a prospective Indian study [27] and 91.4% sensitivity with 94.1% specificity in a subsequent real-world validation across disease stages [28]. Collectively, these findings indicate that AI and augmented intelligence tools have moved beyond proof-of-concept and now constitute clinically actionable technologies with broad translational potential [15].

### 5.2. Comparison with Current Gold-Standard Practice

Traditional glaucoma follow-up still relies on subjective disc-photo reading, noisy perimetry, and sporadic intraocular pressure checks [7]. Meta-analyses put the inter-observer κ for cup-to-disc grading as low as 0.46—only moderate agreement [14]. By contrast, CNNs trained on large fundus datasets reach external, area-weighted κ values of ≈0.88, sharply cutting reader variability and releasing specialist time [13]. Guided progression analysis (GPA) flags change only after three consecutive field losses, whereas a machine learning index that inspects the entire VF sequence detected progression ≈ 1.7 years earlier than the global MD metric in a longitudinal cohort [31]. Sabharwal et al. later showed higher sensitivity and fewer false positives than GPA, although without reporting the lead-time gain [20].

For screening, a smartphone-based offline AI attained 93.7% sensitivity/85.6% specificity, correctly classifying 94.7% of normal cases [27]. A follow-up real-world study confirmed 91.36% sensitivity/94.12% specificity, with false negatives concentrated in early- and moderate-stage eyes and a few advanced cases [28]. Altogether, the evidence indicates that AI delivers its largest incremental benefit where human performance is intrinsically constrained—community triage, early detection, and high-volume monitoring—rather than in tertiary centers already staffed by glaucoma subspecialists [43].

### 5.3. Strengths and Limitations of the Evidence Base

A clear strength of the emerging literature is the steady rise in multicenter, cross-ethnic validation: in our dataset, 18 papers evaluated their algorithms on external cohorts drawn from at least 3 geographic regions. A typical example is the federated Swin Transformer trained across Asia, Europe, and Africa [15], while Rao et al. validated a smartphone classifier in both urban and rural districts of two Indian states [27]. Open-data initiatives are also gaining traction—public releases such as REFUGE2 and the UK Biobank now underpin many benchmarking studies [12].

Important limitations remain. The median sample size per study was only ≈2300 eyes, and fewer than 15% of papers reported calibration metrics or decision curve analyses—findings consistent with Leonard-Hawkhead et al.’s review, which recorded mean adherence to the DECIDE-AI checklist of just 44.6% overall (30.3% for AI-specific items) [23]. Prospective clinical impact evidence is still scant: most systems have been developed and tested on retrospective datasets—including the AI virtual assistant model for primary eye care described by Stuermer et al. [6]—and the few evidence syntheses available on home or remote OCT underline the need for longitudinal studies before widescale adoption [35]. Publication bias probably inflates headline performance, and almost no work has examined cost utility outside Asia. Finally, most algorithms still rely almost exclusively on image inputs, with minimal integration of additional clinical or demographic variables [19]. Across all domains, several weaknesses are consistent: (i) limited attention to calibration and net benefit, (ii) insufficient subgroup analyses (ethnicity, myopia, disc size, and age), (iii) poor replicability across vendors and sites, (iv) scarce evidence of clinical and economic impact, and (v) incomplete reporting relative to DECIDE-AI and TRIPOD-AI. Consequently, while high AUROC values were necessary, we did not consider them sufficient. We placed greater weight on studies with external validation, explicit uncertainty estimates, and clinically relevant comparators.

Recognizing that economic feasibility is highly context-dependent, we analyzed cost-effectiveness alongside adoption barriers across diverse healthcare settings.

### 5.4. Implementation Barriers Mapped to the NASSS Framework

Using the non-adoption, abandonment, scale-up, spread, and sustainability (NASSS) lens, we identify seven categories of risk [51].

Using the NASSS framework, we identified seven categories of challenges that shape adoption and scale-up. At the **condition level**, glaucoma presents substantial complexity: it includes heterogeneous phenotypes—from primary open-angle to narrow-angle, pseudo-exfoliative, and normal-tension glaucoma—making it unlikely that a single model will suit all clinical presentations [12].

The **technology dimension** is equally demanding. Under the EU AI Act, medical AI systems are classified as high-risk devices and must operate within a quality management system that enforces structured risk management, comprehensive technical documentation (including data lineage and validation metrics), automatic activity logs, transparency, and post-market surveillance [24].

From a **value perspective**, adoption depends on demonstrating clear cost-effectiveness. A micro-costing study in rural China estimated an ICER of around USD 1100 per QALY for AI-assisted screening—well below local willingness-to-pay thresholds—yet dedicated reimbursement codes for these systems are still scarce [5].

In terms of **user adoption**, there is limited evidence on how saliency maps influence clinician trust. Current systematic reviews warn that poorly validated visual explanations may create a false sense of security rather than genuine interpretability [18].

At the **organizational level**, GPU capacity, cybersecurity, and workflow redesign drive substantial upfront costs. Smaller clinics often favor cloud-based solutions, but these raise concerns about data sovereignty and vulnerability to adversarial attacks. Emerging approaches, such as federated learning combined with blockchain, aim to address these issues [52].

The **wider context** remains fluid: in the U.S., liability discussions have drawn analogies to computer-aided radiology, while European case law is still unsettled, leaving ambiguity around ultimate responsibility for errors [19].

Finally, **sustainability** requires continuous post-market surveillance for class IIa software in the EU. This includes automated monitoring dashboards, routine bias testing, and transparent version control to track updates and performance drift throughout the product lifecycle [19,24].

### 5.5. Gaps and Priorities for Future Research

- Randomized controlled trials (RCTs). Large pragmatic randomized trials of AI-based glaucoma triage have not yet begun. Multicenter studies with thousands of participants from different regions will be needed to assess effects on vision-related quality of life. In the meantime, smaller investigator-initiated RCTs can compare AI-guided escalation with usual care in fast progressors [16].
- Multimodal fusion. Future multimodal fusion strategies—including corneal biomechanics, OCT angiography, and IOP telemetry—could enhance predictive accuracy beyond the current AUC of 0.832 achieved by Koornwinder et al. using RNFL OCT and EHR data [42].
- Fairness auditing and bias mitigation. Few papers publish stratified metrics by ethnicity, axial length, or disc size. Recent ethics commentaries call for routine bias audits and exploration of counterfactual or reweighting techniques [19].
- Economic and environmental sustainability. Although not directly addressed, lifecycle analyses comparing cloud inference with edge devices could complement privacy-preserving frameworks by supporting environmentally sustainable AI deployment in ophthalmology [52].
- Human–AI interaction science. Eye tracking reveals that expert gaze behavior on OCT reports can inform AI model development and validation, enhancing interpretability and educational value [51]. This supports the need for rigorous interface testing and user-centered design [41,53].
- Regulatory science. Under the EU AI Act, any substantial modification to a high-risk AI system triggers a renewed conformity assessment, effectively enforcing a locked release cycle and a formal change control plan to accompany each update [24].

### 5.6. Limitations of This Review

We restricted the search to English-language, peer-reviewed articles, so influential work published in Chinese, Spanish, or other languages may have been missed. Grey literature and preprints were excluded to preserve evidentiary robustness, but that choice inevitably delays coverage of emerging techniques, such as diffusion models and graph transformers. This paper constitutes a narrative review and does not adhere to PRISMA guidelines for systematic reviews or meta-analyses. We have not preregistered a protocol or conducted a pooled quantitative synthesis, and hence, the selection and interpretation of research have been vulnerable to subjectivity, despite independent screening and predefined qualifying criteria. Because this is a narrative synthesis, no pooled meta-analysis was undertaken, and variation in outcome metrics (e.g., image-level AUC vs. patient-level accuracy) further limits direct quantitative comparison across studies. Finally, the search window closed in July 2025, so developments presented at meetings later in 2025, therefore, fall outside the scope of this review. We focused on identifying consistent patterns across the literature. Notably, diagnostic AUCs tend to cluster in the 0.90–0.97 range in both fundus and OCT studies, with further improvements observed when multimodal or longitudinal approaches are applied. This method provides a structured, evidence-based overview while still accounting for the methodological variability that characterizes the field.

### 5.7. Clinical and Policy Implications

In today’s clinical practice, artificial intelligence acts primarily as a “second reader”: it highlights suspicious images and supports the decision to refer to a specialist, without replacing medical judgment. In confirmation of this, Akkara and Kuriakose recalled the fundus-on-phone project, in which an algorithm installed on a smartphone achieved 95.8% sensitivity and 80.2% specificity in screening for diabetic retinopathy—values considered suitable for triage. The same authors also showed that the heat maps produced with the Integrated Gradients technique make the algorithm’s reasoning more comprehensible and increase the evaluators’ confidence in its results [21].

### 5.8. Summary

Artificial and augmented intelligence tools have progressed from laboratory proofs of concept to clinically piloted systems that detect glaucoma earlier, predict progression more accurately, and standardize management pathways [15]. Over the coming decade, priorities include (i) large-scale, multi-ethnic validation, (ii) prospective outcome trials, (iii) mandatory bias auditing, and (iv) regulatory frameworks that balance safety with innovation. When these prerequisites are met, AI will not replace glaucoma specialists—rather, it will extend their reach and precision, especially in resource-constrained environments [24].

In summary, the growing body of evidence shows that AI in glaucoma has moved from proof-of-concept to real clinical potential. At the same time, it makes clear where the field needs to go next: broader validation across diverse populations, prospective outcome trials, systematic approaches to bias detection, and regulatory models that can adapt as the technology evolves. These points are developed in more detail in Section 6 (Conclusions and Future Directions). Key studies discussed in the Discussion are summarized in Table 5.

## 6. Conclusions and Future Directions

Glaucoma’s silent, irreversible vision loss calls for tools that exceed human limits in early detection and longitudinal monitoring. Over the past decade, artificial—and, recently, augmented—intelligence has progressed from laboratory curiosity to clinically evaluated technology able to (i) capture subtle structural changes of the optic nerve, (ii) predict functional deterioration months before it is measurable at the slit-lamp, (iii) streamline and standardize referral or triage decisions, and (iv) project specialist expertise into underserved areas through teleophthalmology [13,20,27]. Deployment at scale rests on four pillars: (1) Diversity, achieved via training and validation on increasingly multi-ethnic and geographically varied datasets, such as those used in the recent smartphone AI program [27]. (2) Transparency, delivered by interpretable overlays and calibrated confidence scores that nurture clinician trust and delineate liability [18,22]. (3) Regulation, with adaptive algorithm change protocols and post-market surveillance able to intercept performance drift [24]. (4) Partnership, because the greatest value of AI is to augment—not replace—expert judgement, a goal that demands new competences in ophthalmology curricula [16].

Looking ahead, continual multimodal learning—building on current fusion models that combine EHR and OCT data [42]—could benefit from additional inputs, such as OCT angiography, corneal biomechanics, genomics, and real-time IOP telemetry [12]. Federated, privacy-preserving consortia promise to mitigate bias, while real-world evidence from learning health systems will link model performance to outcomes [15,43]. Prospective clinical trials will be needed to verify whether AI-based triage systems effectively reduce the progression of ocular diseases and related healthcare costs. If such trials demonstrate robust clinical and economic benefits, the results could facilitate regulatory approval and reimbursement pathways; conversely, any signs of harm or inequity would require a review of the algorithms and the introduction of further safeguards [19]. In short, properly validated AI can enable earlier intervention, personalized therapy, and more equitable access—shifting glaucoma care from reactive to proactive [5,7,8]. Realizing this vision demands sustained collaboration among clinicians, computer scientists, ethicists, regulators, and, crucially, patients. By embracing AI as a collaborative partner and committing to rigorous oversight, the ophthalmic community can harness technology to safeguard sight worldwide.

## Figures and Tables

**Table 1:** Databases consulted, search terms, and eligibility criteria (inclusion and exclusion) used to identify studies on AI and augmented intelligence in glaucoma.

Aspect	Description
Databases searched	PubMed/MEDLINE, Scopus, IEEE Xplore
Timeframe	1 January 2019–1 July 2025
Search terms	glaucoma, artificial intelligence, machine learning, deep learning, augmented intelligence, OCT, fundus, visual field, decision support, teleophthalmology (combined with Boolean operators)
Inclusion criteria	Articles published in peer-reviewed journals

**Table 2:** Performance metrics and contextual features of representative AI models in glaucoma management.

Study (Year)	Model Type	Input Modality	Primary Task	Performance	Advantages	Limitations
Liu et al. (2019) [13]	GD-CNN (ResNet-based)	Fundus photographs	Detection of glaucomatous optic neuropathy	AUC 0.996 (internal); 0.995–0.987 (clinical); 0.964 (population); 0.923 (multi-ethnic). Sens 93.6–96.2%; Spec 95.6–97.7% (drops to Sens 87.7%, Spec 80.8% on multi-ethnic; 82%/70% on low-quality images)	Very large (241,032) training set; multiple external validations; online learning module	Needs centered, good-quality fundus images; performance falls on multi-ethnic and low-quality datasets
Sabharwal et al. (2023) [20]	CNN–LSTM	Serial SAP deviation maps	Detection of VF worsening	AUC 0.94 (full data) vs. 0.82 mixed effects, *p* < 0.001; AUC 0.78 with six VFs removed	Spatiotemporal modeling; robust with fewer tests	Single-center; ≥7 reliable VFs; external validation pending
Wang et al. (2024) [25]	Vision transformer + FC classifier	Baseline VF 24-2 grid + OCT RNFL grid + clinical and demographic data	Forecast need for glaucoma surgery	AUROC 0.92 (0–3 months); ≥0.85 up to 2 years; 0.76 at 4–5 years	Single-visit risk stratification; SHAP interpretability highlights IOP, MD, and RNFL	Tertiary center cohort; performance declines > 3 years; requires both OCT and VF
Chen et al. (2023) [16]	Deep learning CDSS	EHR data + visual field metrics	Usability of a CDSS displaying predicted MD (from OCT) + clinical data	Likert trust = 3.27, Likert utility = 3.42, Likert willingness to decrease VF = 2.64, SUS = 66.1 ± 16.0 (43rd percentile) Tendency to maintain management in mild cases and to escalate in advanced cases, without formally measuring percentage changes from a predefined plan	Real-world integration; enhances shared decision-making	Single center, n = 10 clinicians/6 cases, demographically non-diverse sample (all white patients), usability study (not clinical outcomes)
Ran et al. (2024) [15]	Federated 3D CNNs (FedProx)	**Volumetric OCT scans** (7 centers)	Glaucoma detection via FL	Accuracy per center 78–98%; two unseen test sets 81–88%; FL not inferior to the centralized model	Preserves data privacy; leverages multi-site OCT without data sharing	Needs FL infrastructure; performance still variable across sites; OCT required

**Table 3:** Summary of representative studies on AI and augmented intelligence applied to glaucoma. Each entry reports the model type, input modality, primary task, main performance outcomes, and key advantages and limitations, highlighting the diversity of approaches and their current stage of clinical readiness.

Study (Year)	Model Type	Input Modality	Primary Task	Performance	Advantages	Limitations
**Liu et al. (2019)** [13]	ResNet-based CNN	Fundus photographs	Detection of glaucomatous optic neuropathy	AUC up to 0.996 (internal); 0.964 (population); 0.923 (multi-ethnic). Sensitivity 93–96%; specificity 95–97% (drops to ~82/70% on low-quality images).	Very large training set (>240 k images); multiple external validations; online module for continuous learning.	Requires high-quality, centered fundus images; performance falls in multi-ethnic and low-quality datasets.
**Medeiros et al. (2019)** [26]	ResNet-34 CNN	Fundus photographs → OCT regression	Cross-modal prediction of RNFL thickness	r ≈ 0.83; MAE ≈ 7 µm.	First demonstration of cross-modal learning; bridges fundus and OCT.	Single center; requires further external validation.
**Sabharwal et al. (2023)** [20]	CNN–LSTM	Serial SAP deviation maps	Detection of visual field worsening	AUC 0.94 vs. 0.82 (linear model); robust even with fewer tests (AUC 0.78).	Spatiotemporal modeling; detects change earlier with limited data.	Single center; requires ≥ 7 reliable VFs; external validation pending.
**Wang et al. (2024)** [25]	Vision transformer + classifier	Baseline VF + OCT RNFL + demographics	Forecast need for glaucoma surgery	AUROC 0.92 at 3 months; ≥0.85 up to 2 years; 0.76 at 4–5 years.	Single-visit risk stratification; interpretable (SHAP values highlighted IOP, MD, and RNFL).	Cohort from a tertiary center; performance drops at longer horizons; requires both VF and OCT.
**Rao et al. (2024)** [27]	Offline CNN	Smartphone fundus camera	Referable glaucoma screening	Sensitivity 93.7%; specificity 85.6%; TN rate 94.7%.	Works offline; feasible in low-resource settings.	Performance depends on image quality; prospective trial but limited geography.
**Senthil et al. (2025)** [28]	Offline CNN	Smartphone fundus camera	Real-world screening across glaucoma stages	Sensitivity 91.4%; specificity 94.1%.	Specialist-confirmed cohort; good performance across stages.	False negatives in early/moderate stages; limited to Indian clinics.
**Ran et al. (2024)** [15]	Federated 3D CNNs (FedProx)	Multicenter OCT scans (7 sites)	Glaucoma detection via federated learning	Accuracy 78–98% per center; 81–88% on unseen test sets; FL non-inferior to centralized model.	Preserves privacy; leverages data across centers without sharing raw images.	Requires federated infrastructure; variable performance across sites.
**Chen et al. (2023)** [16]	Deep learning CDSS	EHR data + VF metrics	Usability of decision support for VF progression	Trust Likert 3.27; usefulness 3.42; SUS 66.1 (43rd percentile).	Real-world integration; supports shared decision-making.	Pilot usability study; only 10 clinicians, 6 cases; not clinical outcomes.
**Tian et al. (2024)** [22]	U-Net trained on expert gaze	OCT glaucoma reports with eye tracking	Prediction of clinically relevant regions	Precision 0.72; recall 0.56; F1 0.61.	First use of expert gaze to guide saliency; enhances interpretability.	Still experimental; small dataset; requires validation for clinical use.

**Table 4:** Cross-domain synthesis of strengths, weaknesses, risks, maturity of evidence, and future priorities in AI for glaucoma.

Domain	Strengths	Weaknesses	Typical risks	Evidence maturity (TRL)	Next steps	Key Refs.
**3.1 Diagnostic imaging (fundus and OCT)**	Consistently high diagnostic accuracy (AUC ~0.90–0.97); validated across large datasets; newer models (multi-task CNNs, ViTs, and 3D U-Nets) are more efficient; multimodal approaches cut false positives.	Calibration rarely reported; metrics often image- rather than patient-based; performance falls on multi-ethnic or low-quality datasets; little attention to challenging phenotypes (NTG, high myopia, and small discs); poor vendor generalizability.	Spectrum bias from enriched case series; domain shift with new devices; risk of data leakage.	TRL 6–7: solid retrospective, some multicenter; few prospective trials with clinical endpoints.	Large prospective multi-vendor studies; patient-level metrics; decision curve analyses; subgroup robustness; explicit domain-shift protocols.	[7,9,11,14,23,26,29,30]
**3.2 Progression prediction and monitoring**	Longitudinal models detect worsening earlier than linear methods; good sensitivity even with fewer tests; single-visit multimodal models predict outcomes, such as need for surgery; some provide individualized risk curves.	Mostly retrospective, often single-center; definitions of progression heterogeneous; little use of lead-time or net-benefit analyses; risk of temporal data leakage; limited testing in complex phenotypes (PXF, PACG, and NTG).	Temporal leakage; censoring bias; selective follow-up.	TRL 5–6: strong longitudinal cohorts but very few intervention studies.	Pragmatic RCTs on follow-up schedules and treatment impact; adoption of decision curve analysis; harmonized progression definitions; analyses in complex phenotypes.	[20,31,32]
**3.3 Clinical decision support and LLMs**	CDSS judged useful, especially in complex cases; LLMs perform at subspecialist level in knowledge tasks; first prototypes integrated.	Small samples; usability studies dominate over outcome-driven trials; lack of calibration and uncertainty estimates; risk of hallucination; no standardized human-in-the-loop workflows; medico-legal concerns.	Over-reliance on uncalibrated output; drift in EHR data; liability uncertainties.	TRL 4–5: promising prototypes, but impact evidence still limited.	Clinical trials measuring process and patient outcomes; explicit uncertainty and calibration; governance for medico-legal accountability; periodic audits.	[16,17,33,34]
**3.4 Teleophthalmology and remote monitoring**	Offline/on-device algorithms with high sensitivity and specificity; feasibility shown in low-resource settings; home OCT highly concordant with in-clinic OCT; early multimodal models forecast rapid worsening.	Evidence geographically concentrated (mainly India); performance tied to image quality; short follow-up; limited data on adherence; little cost-effectiveness outside Asia.	Workload redistribution; image quality variability; digital divide; self-selection of adherent users.	TRL 6: early prospective/real-world studies, but community-level outcomes still lacking.	Multicenter community trials; cost-effectiveness in different regions; structured referral pathways; long-term adherence data.	[5,27,28,35,36]
**3.5 Explainable and federated AI**	Saliency and attention maps often overlap with clinically meaningful regions; federated learning preserves privacy and achieves non-inferior accuracy; first conceptual frameworks for privacy-preserving deployment.	No standard metrics for XAI; saliency maps not proven to improve trust or accuracy; FL infrastructure demanding; regulatory pathway for continuously updated models unclear; little evidence on clinical impact.	Misleading saliency; inter-site heterogeneity; cybersecurity threats; weak version control.	TRL 4–6: proof-of-concept strong, but long-term maintainability and impact evidence thin.	Shared benchmarks for XAI; routine bias audits; studies on maintainability and change control (EU AI Act and FDA GMLP); scalable FL implementations.	[15,18,19,22,24]

**Table 5:** Key studies discussed in the Discussion Section, with main contributions, limitations, and future directions in AI for glaucoma care.

Theme	Study (Year)	Contribution	Limitations	Future Directions
**Diagnosis**	Liu et al. (2019) [13]	CNN on >240 k fundus photos, AUROC ~0.96–0.99; robust across datasets.	Performance drops in low-quality and multi-ethnic datasets.	Larger, more diverse cohorts to improve generalizability.
	Nguyen et al. (2025) [9]	Head-to-head: AI matched/surpassed 13 clinicians in detecting referable glaucoma.	Tested retrospectively; generalizability beyond safety-net populations unclear.	Prospective validation in routine care.
	Medeiros et al. (2019) [26]	Cross-modal CNN predicting OCT RNFL thickness from fundus images (r ≈ 0.83).	Proof of concept; not validated in real-world settings.	Extend to multimodal integration and prospective testing.
**Progression**	Sabharwal et al. (2023) [20]	CNN-LSTM detected VF worsening (AUC 0.94), outperforming mixed-effects models.	Single center; requires ≥ 7 reliable VFs.	Multicenter validation and inclusion of fewer tests.
	Yousefi et al. (2018) [31]	Machine learning VF progression index detected deterioration ~1.7 years earlier.	Retrospective; limited external validation.	Incorporate into clinical workflows to reduce detection lag.
**Decision Support and LLMs**	Chen et al. (2023) [16]	GLANCE CDSS dashboard judged usable; supported but did not replace management.	Usability study only; small clinician sample.	Larger trials assessing impact on patient outcomes.
	Huang et al. (2024) [17]; Delsoz et al. (2023) [33]	GPT-4 and ChatGPT matched or surpassed subspecialists on clinical cases.	Requires expert oversight; limited datasets.	Define safe integration of LLMs into decision-making.
	Zhang et al. (2024) [34]	GPT-4o comparable to experienced ophthalmologists in differential diagnosis.	Lower absolute accuracy vs. clinicians; single center.	Evaluate LLM utility in complex cases with multicenter data.
**Teleophthalmology**	Rao et al. (2024) [27]	Smartphone-based AI achieved 93.7% sensitivity, 85.6% specificity.	Tested in Indian cohorts only.	Broader validation in community screening programs.
	Senthil et al. (2025) [28]	Real-world study confirmed 91.4% sensitivity, 94.1% specificity.	False negatives in early/moderate stages.	Improve sensitivity for early disease.
	Dolar Szczasny et al. (2024) [35]	Systematic review: home OCT devices highly concordant with in-clinic OCT.	Pilot studies, short follow-up.	Longitudinal trials to test real-world adoption.
**Explainable and Federated AI**	Ran et al. (2024) [15]	Federated learning across 7 centers preserved privacy, accuracy up to 98%.	Infrastructure heavy; performance varied across sites.	Scalable FL frameworks for clinical use.
	Wong et al. (2024) [18]	Review: saliency maps do not reliably improve trust or accuracy.	Low empirical support; risk of misleading overlays.	Standardized metrics for XAI evaluation.
	Teo et al. (2025) [52]	FL + blockchain framework proposed for adversarial protection.	Conceptual; not yet clinically tested.	Pilot implementations in resource-constrained settings.
	Akerman et al. (2023) [53]	Eye tracking revealed expert gaze patterns informative for AI training.	Exploratory; small sample.	Human–AI interaction science to refine interpretability.
**Reporting and Evidence Quality**	Leonard Hawkhead et al. (2025) [23]	Systematic review: DECIDE-AI adherence ~45% overall, ~30% for AI-specific items.	Highlights poor reporting standards.	Enforce standardized reporting for AI clinical trials.
	Stuermer et al. (2025) [6]	Review: AI virtual assistants in primary care raise cost and workflow concerns.	No outcome studies; mainly conceptual.	Prospective health–economic evaluations.

## Data Availability

No new data were created or analyzed in this study. Data sharing is not applicable to this article.
